# Increased plasma imatinib exposure and toxicity in chronically treated GIST patients with SARS-CoV-2 infection: a case series

**DOI:** 10.3389/fimmu.2024.1441620

**Published:** 2024-10-09

**Authors:** Sara Gagno, Bianca Posocco, Marco Orleni, Eleonora Cecchin, Arianna Fumagalli, Michela Guardascione, Angela Buonadonna, Jerry Polesel, Fabio Puglisi, Giuseppe Toffoli, Erika Cecchin

**Affiliations:** ^1^ Experimental and Clinical Pharmacology, Centro di Riferimento Oncologico di Aviano (CRO), IRCCS, Aviano, Italy; ^2^ Department of Medical Oncology, Centro di Riferimento Oncologico di Aviano (CRO), IRCCS, Aviano, Italy; ^3^ Unit of Cancer Epidemiology, Centro di Riferimento Oncologico di Aviano (CRO) IRCCS, Aviano, Italy; ^4^ Department of Medicine, University of Udine, Udine, UD, Italy

**Keywords:** imatinib, TDM, pharmacogenetics, COVID-19, case report

## Abstract

**Introduction:**

Inflammatory factors released during severe coronavirus disease-19 (COVID-19) caused by acute respiratory syndrome coronavirus 2 (SARS-CoV-2) are known to influence drug exposure, but data on the effect of mild infection are few. Here we describe for the first time an increase in plasma imatinib and norimatinib concentrations observed in a series of 5 patients treated with imatinib for gastrointestinal stromal tumor (GIST) after mild COVID-19.

**Methods:**

The patients were undergoing routine therapeutic drug monitoring (TDM) and pharmacogenetic (PGx) analyses of polymorphisms in genes involved in imatinib metabolism and transport (*CYP3A4*, *CYP3A5*, *ABCB1*, and *ABCG2*) when SARS-CoV-2 infection occurred. Imatinib and its active metabolite norimatinib concentrations were determined at C_trough_ using a validated LC-MS/MS method. PGx analyses were performed by KASP genotyping assays on a Real-Time PCR system. All patients received imatinib 400 mg/day. Case 1 was prospectively monitored. Cases 2-5 were identified retrospectively.

**Results:**

On average, imatinib C_trough_ increased significantly by 70% during COVID-19, whereas norimatinib showed a 44% increase compared with pre-COVID-19 levels. Elevated plasma imatinib concentrations persisted up to 6 months after infection remission. In 3 cases, this increase reflected the occurrence or worsening of imatinib side effects.

**Conclusion:**

This case-series highlights the clinical impact of SARS-CoV-2 infection on the management of patients with GIST treated with imatinib.

## 1 Introduction

Inflammation is a complex biological response to harmful stimuli such as pathogens, damaged cells, or irritants, and it plays a pivotal role in the body’s immune defense mechanisms. While inflammation is essential for protecting and healing the body, it can also significantly impact various physiological processes, including drug metabolism. One of the critical systems affected by inflammation is the cytochrome P450 (CYP) enzyme family, a group of enzymes responsible for the metabolism of a vast array of drugs and endogenous compounds, affecting their pharmacokinetic profiles and, consequently, their efficacy and toxicity. Inflammatory cytokines released during acute or chronic inflammatory status as interleukin-6 (IL-6) and tumor necrosis factor-alpha (TNF-α) are known to downregulate CYP450 enzyme activity, leading to a reduction in the metabolism of a large number of drugs (1) with consequences on drug exposure and clinical outcome. The massive release of cytokines that occurs during severe coronavirus disease-19 (COVID-19, caused by SARS-CoV-2 infection), also known as “cytokine storm”, was shown to influence the drug metabolism by modulating the expression and activity of specific cytochromes (2). In addition, the direct liver damage caused by severe COVID-19 may influence the pharmacokinetics of drugs by reducing their clearance and increasing their bioavailability (3).

**Figure 1:**
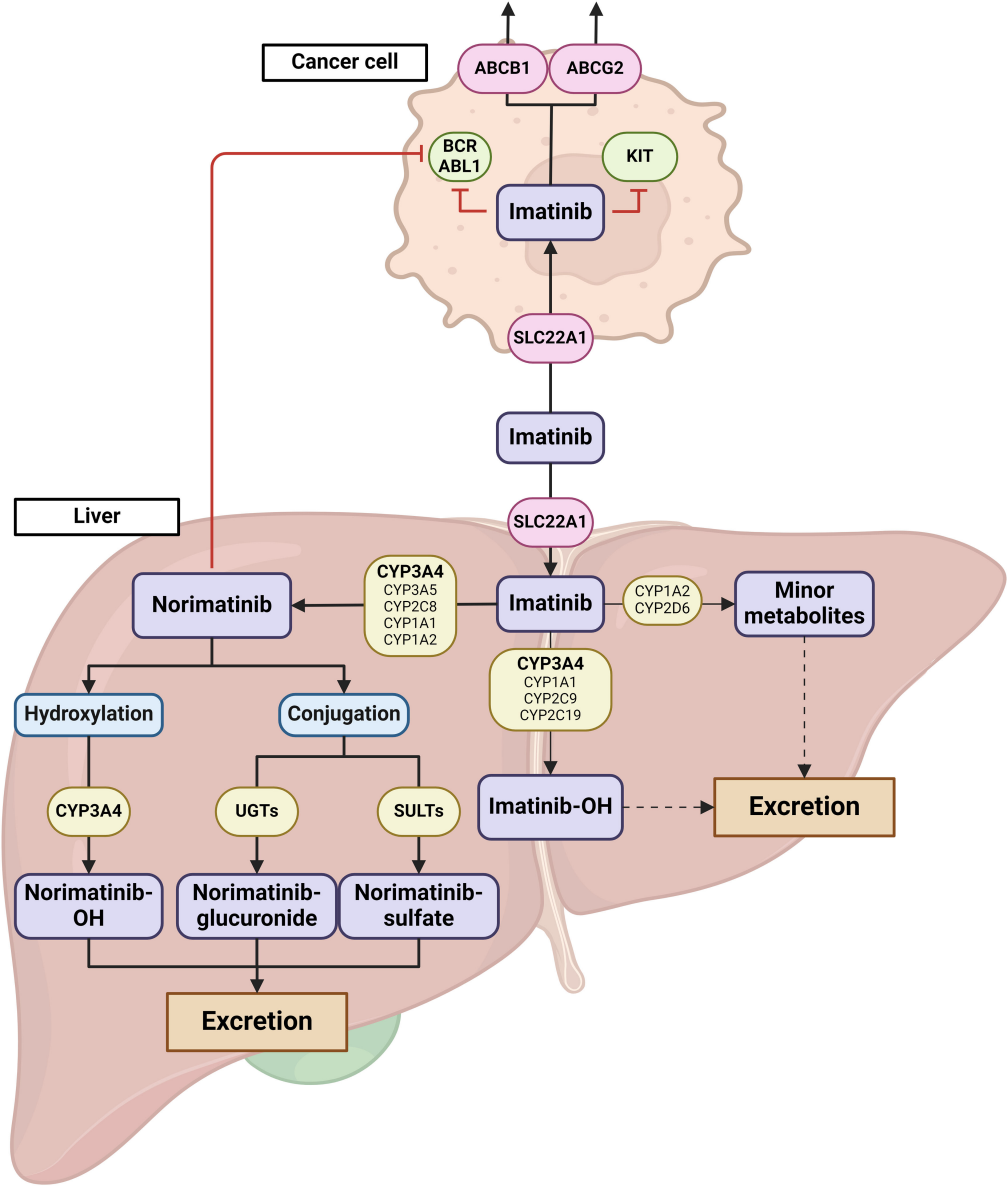
Graphical representation of imatinib metabolism and transport. Imatinib uptake is facilitated by SLC22A1 transporters, both in the liver and in cancer cells. The primary metabolic pathway of imatinib involves demethylation mediated by CYP3A4, resulting in the formation of N-desmethylimatinib (norimatinib). While other CYP450 enzymes, including CYP3A5, CYP2C8, CYP1A1, and CYP1A2, also participate in this process, their roles are relatively minor. Norimatinib, which retains similar activity to imatinib, undergoes further metabolism either through CYP3A4-mediated hydroxylation or through conjugation reactions (glucuronidation or sulfonation). These more hydrophilic metabolites are then excreted via urine or feces. Additionally, imatinib can be metabolized through minor pathways involving CYP3A4, CYP3A5, CYP1A1, CYP1A2, CYP2C9, CYP2C19, and CYP2D6 leading to hydroxylated products or the formation of imatinib N-oxide metabolites, which are also excreted through urine and feces. In the figure, imatinib transporters are represented by red circles, while metabolizing enzymes are shown in yellow boxes. Major metabolic pathways are highlighted with thick arrows, while minor pathways are depicted with thin arrows. Dashed arrows indicate minor metabolic routes not shown in the figure. The primary enzymes involved in each metabolic step are indicated in bold text. Created with BioRender.com

Imatinib, is a selective tyrosine kinase inhibitor (TKI) used in chronic myeloid leukemia (CML) and gastrointestinal stromal tumors (GIST). It is metabolized primarily by CYP3A4 into its equipotent metabolite norimatinib. Norimatinib undergoes further metabolism primarily through hydroxylation, predominantly mediated by CYP3A4. Additionally, it can enter phase II metabolism, where it is conjugated with glucuronic acid via UGT enzymes or with sulfate via SULT enzymes (4). These conjugated metabolites are subsequently excreted from the body through urine and feces. While other cytochrome P450 enzymes (CYP1A1, CYP1A2, CYP2D6, CYP2C9, and CYP2C19) contribute to imatinib metabolism, their roles are relatively minor compared to CYP3A4 (4). Imatinib was also shown to be actively transported through cell membranes by ABC-proteins (i.e., P-glycoprotein P, P-gp) encoded by *ABCB1* gene and Breast Cancer Resistance Protein (BCRP) encoded by *ABCG2* gene (5) (**Figure 1**). Large inter-individual variability in plasma exposure to imatinib, which is orally administered at a fixed dose, is reported to affect both efficacy and toxicity. Current guidelines recommend a target steady-state minimum plasma concentration (C_trough_) greater than 1100 ng/mL for treatment efficacy in GIST, while a C_trough_ exceeding 3000 ng/mL has been associated with severe toxicity (6). Differences in pharmacogenetic profile and interactions with co-medications reportedly affect imatinib exposure (7, 8). Given the crucial role of CYP3A4 and other CYP450 family members in imatinib metabolism, any variation in their activity can result in altered exposure to the drug. In this case series we describe the observed effect of COVID-19 with mild symptoms on imatinib exposure and toxicity in 5 patients with GIST.

## 2 Materials and methods

### 2.1 Methodology of patients’ selection

This study includes patients treated with imatinib affected by GIST and selected from the cohorts of two clinical studies approved by the local ethics committee (Comitato Etico Unico Regionale del Friuli Venezia Giulia - CEUR) and conducted in accordance with the principles of the Declaration of Helsinki in its latest version: 1) “Pilot study to evaluate the feasibility of an innovative approach to monitor patients with gastrointestinal stromal tumor treated with imatinib” (protocol ID: CRO-2017-19, EuDRACT: 2017-002437-36, approval date 21/12/2017, parere CEUR-2017-Sper-125-CRO) and 2) CRO–Aviano integrated pharmacological counselling program (protocol ID: CRO 2022-14, approval date April 12, 2022; Parere-CEUR-2022-Os-65). All the patients were of European descent. Written informed consent was obtained from the patients for the participation in the study and for the publication of the results. Case 1 was prospectively monitored. Cases 2-5 were retrospectively identified by mining the study database to identify other patients who may have experienced COVID-19 while participating in the trial. Selection for the present analysis was based on the following criteria: 1. (self-reported) antigen test confirming COVID-19 during imatinib treatment, 2. at least two blood samples for imatinib quantification in the period before SARS-CoV-2 infection, 3. at least one blood sample within 6 months after COVID-19. Patients with no clear diagnosis of COVID-19 or having the first blood sample for imatinib quantification taken more than 6 months after COVID-19 recovery were excluded. Based on these criteria, only 4 additional GIST patients were retrospectively identified from an “active” case population (meaning the patients still alive and actively participating in the study at the time of patients selection) of 32 patients treated with imatinib.

### 2.2 Data collection

**Figure 2:**
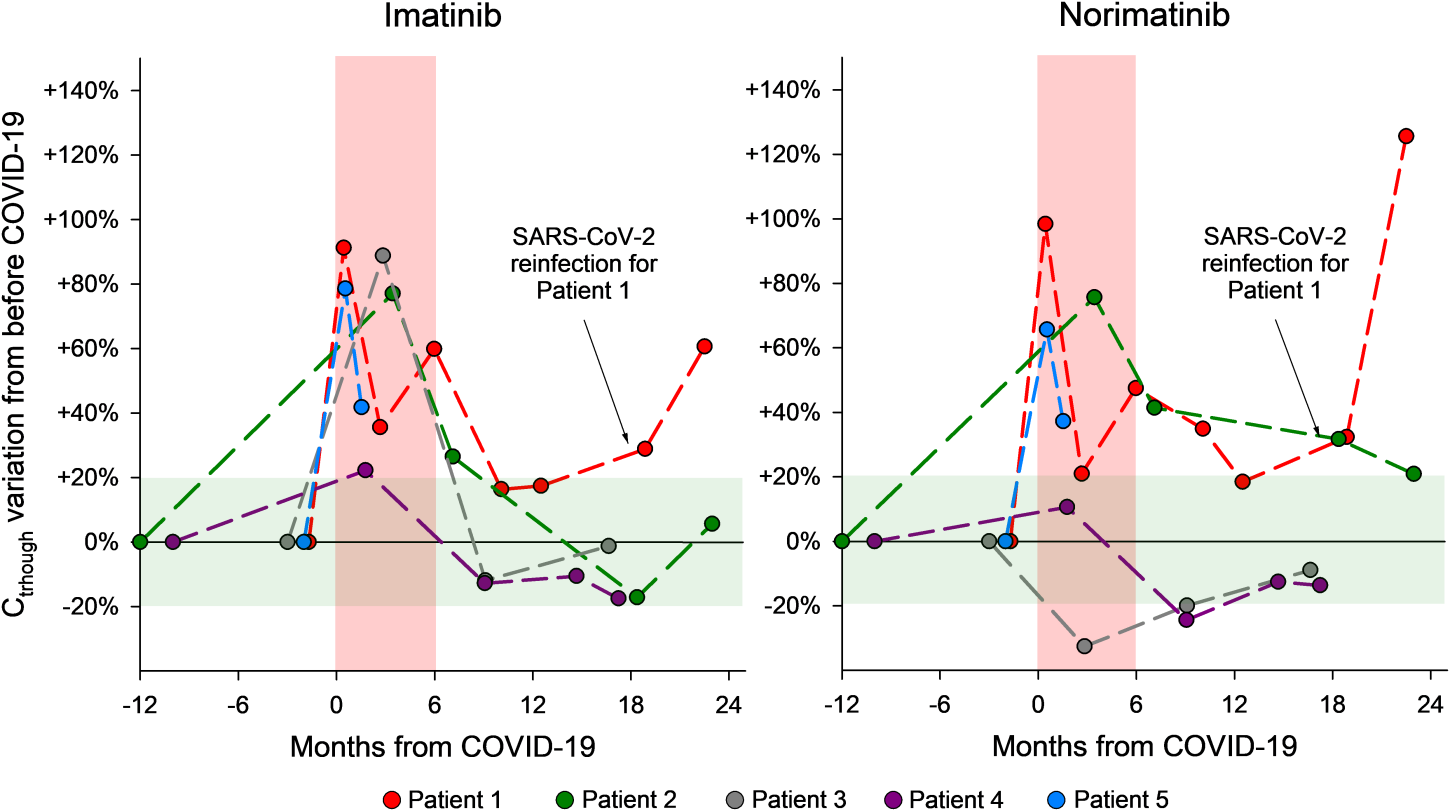
Graphical representation of the changes in plasma imatinib and norimatinib levels during the monitoring period for the 5 GIST cases described. The pre-COVID-19 values were calculated as the average of two blood samples taken before COVID-19 and set as baseline values. The concentration variations that occurred during the acute COVID-19 phase and afterwards are reported as a percentage difference compared with the baseline (pre-COVID-19) value. The red area indicates a period of 6 months since the SARS-CoV-2 infection; the green area shows acceptable concentration fluctuations according to the limits of the analytical method (± 20%).

Relevant demographic information, clinical data, patients’ characteristics (comorbidities, co-medications, DDIs), and life-style habits potentially affecting drug exposure (cigarette smoking, alcohol intake), for the 5 patients here described are reported in **Table 1**, while the results of the laboratory tests performed are reported in **Table 2**. A timeline of patients’ samplings and imatinib and norimatinib C_trough_ variations is displayed in **Figure 2**.

**Table 1:** Patient’s clinical-demographic characteristics.

	Case 1	Case 2	Case 3	Case 4	Case 5
Demographic data					
Sex	F	M	M	M	M
Age at COVID-19 infection (years)	50	57	71	45	82
Clinical data					
GIST site	Gastric	Abdominal	Small bowel	Gastric	Small bowel
Year of diagnosis	2019	2003	2002	2010	2016
Metastasis at diagnosis	Liver	No	No	No	No
COVID-19 data					
Date of COVID-19 infection	March 2022, January 2024	January 2022	October 2022	October 2022	December 2023
COVID-19 vaccinations before infection	3 doses	2 doses	3 doses	2 doses	3 doses
Toxicity during COVID-19	Muscle cramps G2, periorbital edema G2	None	CPK increase G1	None	Periorbital and lower limb edemas G1, gynecomastia G2
Life-style habits					
Cigarette smoke	No	No	No	No	No
Alcohol intake	No	Sporadic	1-2 glasses/day	Sporadic	No
Co-morbidities					
Co-morbidities type	None	Chronic renal failure, diabetes, hypertension, depression, dyslipidemia, hepatic steatosis, esophagitis, hiatal hernia	Hypertension, dyslipidemia, ischemic heart disease, hyperuricemia, chronic renal failure in single kidney patient	None	Resected prostate cancer (2013), resected renal carcinoma (2017)
Co-medications	None	Pantoprazole, ramipril, atorvastatin, fibrinogen, venlafaxine, insulin lispro, insulin glargine, pioglitazone, canagliflozin, fenofibrate	Pantoprazole, ramipril, rosuvastatine, ezetimibe, acetylsalcycilic acid, clopidogrel, isosorbide dinitrate, allopurinol	None	None
Relevant drug-drug interactions	NA	Hypoglycemic agents (6 interactions with ↑ risk of hypoglycemic effects) Imatinib/atorvastatin (possible ↑atorvastatin concentration) Fenofibrate/atorvastatin (possible ↑atorvastatin side effects) Pantoprazole/pioglitazone (possible ↑pioglitazione side effects)	Rosuvastatin/Clopidogrel (possible ↑rosuvastatin side effects as myalgia and rhabdomyolysis) Acetylsalicylic acid/clopidogrel (possible ↑ antiplatelet effects and bleeding) Pantoprazole/clopidogrel (possible ↓ concentration of active clopidogrel metabolite, possible risk of impaired clopidogrel effectiveness) Isosorbide dinitrate/ramipril (↑ risk of hypotensive effects) Ramipril/allopurinol (potential ↑ of allergic or hypersensitivity reaction to allopurinol) Acetylsalicylic acid/ramipril (possible ↑ nephrotoxic effects of ramipril, possible ↓ ramipril therapeutic effects)	NA	NA

F, Female; M, male; COVID-19, coronavirus disease 19; GIST, gastrointestinal stromal tumor; G, grade; CPK, creatine phosphokinase.

↑, increase/increased. ↓, decrease/decreased.

**Table 2:** Results of laboratory tests performed for the 5 cases included in the study.

Laboratory tests		Case 1	Case 2	Case 3	Case 4	Case 5
Therapeutic Drug Monitoring						
Imatinib levels (± SD) (ng/mL)	*Pre-COVID-19	659 ± 7	561 ± 38	1419 ± 76	730 ± 130	2064 ± 141
	1st evaluation after COVID-19 (†)	1260 (+90%) 2 day after negativization	994 (+77%) 3 months after negativization	2679 (+89%) 3 months after negativization	893 (+22%) 2 months after negativization	3685 (+79%) 2 weeks after negativization
	**Post COVID-19	770 ± 5	529 ± 91	1326 ± 107	631 ± 26	°2925
Norimatinib levels (± SD) (ng/mL)	*Pre-COVID-19	144 ± 7	162 ± 30	289 ± 25	229 ± 33	332 ± 88
	1st evaluation after COVID-19 (†)	285 (+98%)	285 (+76%)	195 (-33%)	253 (+11%)	550 (+66%)
	**Post COVID-19	182 ± 17	205 ± 12	247 ± 23	190 ± 15	456°
Metabolic ratio	Pre-COVID-19	22%	29%	20%	31%	16%
	1st evaluation after COVID-19	23%	29%	7%	28%	15%
	Post COVID-19	24%	40%	19%	30%	°16%
Pharmacogenetics analyses						
Gene	Variant					
*CYP3A4* (NM_017460.6)	rs35599367 c.522-191C>T (*22)	c.522-191CC	c.522-191CC	c.522-191CC	c.522-191CC	c.522-191CC
*CYP3A5* (NM_000777.5)	See below#	CYP3A5 *1/*1	CYP3A5 *1/*1	CYP3A5 *1/*1	CYP3A5 *1/*3	CYP3A5 *1/*3
*ABCB1* (NM_001348946.2)	rs1128503 c.1236C>T	c.1236CT	c.1236TT	c.1236CT	c.1236TT	c.1236CT
*ABCB1* (NM_001348946.2)	rs1045642 c.3435C>T	c.3435TT	c.3435CT	c.3435CT	c.3435TT	c.3435CT
*ABCB1* (NM_001348946.2)	rs2032582 c.2677T>G>A	c.2677GT	c.2677TT	c.2677GT	c.2677TT	c.2677GT
*ABCG2* (NM_004827.3)	rs2231142 c.421C>A	c.421CC	c.421CC	c.421CC	c.421CA	c.421AA
Inflammation markers						
CRP (reference interval <0.5 mg/dL)	Pre-COVID-19	<0.29 mg/dL	0.56 mg/dL	<0.29 mg/dL	<0.29 mg/dL	NA
	1st evaluation after COVID-19	<0.29 mg/dL	0.78 mg/dL	<0.29 mg/dL	<0.29 mg/dL	NA
	Post COVID-19	<0.29 mg/dL	0.593 mg/dL	<0.29 mg/dL	<0.29 mg/dL	NA
AGP (reference interval 50-130 mg/dL)	Pre-COVID-19	61 mg/dL	100 mg/dL	101 mg/dL	66 mg/dL	NA
	1st evaluation after COVID-19	99 mg/dL	96 mg/dL	71 mg/dL	81 mg/dL	NA
	Post COVID-19	55 mg/dL	98 mg/dL	70 mg/dL	61 mg/dL	NA

SD, standard deviation; CRP, C-reactive protein; NA, not available; AGP, alpha-1 acid glycoprotein.

*The pre-COVID-19 levels of imatinib and norimatinib were estimated as the average levels of two blood samples collected before COVID-19.

**The post-COVID-19 levels of imatinib and norimatinib were estimated as the average levels in blood samples collected when pre-COVID-19 concentrations were restored.

°data from a single sample taken 2 months after COVID-19.

†in brackets is the percentage increase over pre-COVID-19 levels.

Based on the phenotype derived from the following CYP3A5 polymorphisms: rs776746 (*3) c.219-237G>A; rs10264272 (*6) c.624G>A; rs41303343 (*7) c.1035dup.

To establish baseline imatinib and norimatinib plasma levels prior to COVID-19 infection, the mean value from at least two plasma measurements taken before the onset of infection was calculated. This was done in order to account for possible intra-patient fluctuations unrelated to the infection. The value obtained was set as baseline. Drug levels variations during and after COVID-19 are reported as a percentage difference compared with the baseline (pre-COVID-19) value.

### 2.3 Active pharmacology monitoring and laboratory tests

The patients in this case series underwent routine integrated pharmacological counseling, which included imatinib therapeutic drug monitoring (TDM), pharmacogenetic (PGx) analyses of polymorphisms in genes involved in imatinib metabolism (*CYP3A4* and *CYP3A5*) and transport (*ABCB1* and *ABCG2*), as well as an evaluation of potential drug-drug interactions (DDIs). Imatinib and its active metabolite norimatinib concentrations were determined at C_trough_ using a validated LC-MS/MS method. PGx analyses were performed by KASP genotyping assays on a Real-Time PCR system. The polymorphisms selected are reported in **Table 2**. The potential for DDIs was checked using Lexicomp (UpToDate).

C-reactive protein (CRP) and alpha-1-acid glycoprotein (AGP) were quantified in selected samples using a Dimension Vista 1500 intelligent Lab System (Siemens) from frozen plasma.

## 3 Presentation of the cases

### 3.1 Case 1

In 2019, a 47-year-old woman was diagnosed with high-risk GIST of the stomach with liver metastases. At the time of diagnosis, first-line treatment with imatinib 400 mg/day was started with good disease control to date. In November 2020, patient 1 started a periodic integrated pharmacological counseling. Imatinib pre-COVID-19 mean C_trough_ was 659 ± 7 ng/mL. The mean norimatinib/imatinib C_trough_ (metabolic ratio, MR) was 22%. In March 2022, the patient had positive SARS-CoV-2 antigenic tests for 2 weeks, with moderate symptoms. A few days after infection remission, imatinib C_trough_ was found to be increased by ~90% (to 1260 ng/mL) and remained elevated for 6 months (approximately +36% and +60% above the pre-COVID-19 mean exposure). Consistently, the patient reported increased imatinib side effects (grade 2 (G2) muscle cramps and periorbital edema). The MR remained quite stable over the entire monitoring period, with a mean ratio of 22% (19-24%). Pre-infection plasma imatinib levels were restored after approximately one year (mean C_trough_ 770 ± 5 ng/mL). In January 2024, the patient had another episode of mild COVID-19. One week after the first subsequent negative test, imatinib C_trough_ increased again by ~60% compared to the average pre-infection level (1058 ng/mL vs. 659 ng/mL), without worsened side effects. The patient was genotyped for CYP3A4*22, CYP3A5*3, CYP3A5*6, and CYP3A5*7 genetic polymorphisms potentially affecting her drug-metabolizing capacity, but no relevant variant was detected. She was also bearing heterozygous *ABCB1* rs1128503-1236C>T and rs2032582-2677G>T/A polymorphisms, which were not reported to affect drug transport capacity (9). To evaluate the baseline and the potential changes in the inflammatory status of the patient during COVID-19, it was previously suggested to measure the acute phase inflammatory proteins CRP and AGP (10). While the CRP was within the normal range (<0.5 mg/dL) and showed no consistent trend with the concentration fluctuations, the AGP peaked (99 mg/dL) with the highest imatinib concentration, although remaining within the normal range (50-130 mg/dL) (**Table 2**).

### 3.2 Cases 2-5

These patients were all male, aged 57 (case 2), 71 (case 3), 45 (case 4), and 82 (case 5) at the time of SARS-CoV-2 infection and were treated with imatinib 400 mg/day. Cases 2 and 3 had a series of other co-morbidities and were taking several co-medications, while case 5 had a history of two other types of cancer, both resected long before COVID-19 (**Table 1**). Patients’ polypharmacy was almost consistent across the different samples, with only minor changes of no clinical relevance. All the DDIs detected were among drugs used to treat patients’ co-morbidity and had no predicted impact on imatinib plasma levels. Despite the risk evidenced by the DDI analysis, the patients did not self-report an increase in related adverse events. None of the patients was a current or former smoker, nor had unusual alcohol intake.

The imatinib and norimatinib concentrations during and after acute COVID-19 are reported in **Table 2**. For patients 2-4, samples collected 7-9 months after their first post-infection negative tests showed a restoration of pre-COVID-19 imatinib exposure. At his last follow-up (2 months after infection), patient 5 had still not achieved his pre-COVID-19 levels (**Figure 1**). Case 3 and 5 experienced toxicity, detailed in **Table 1**.

Considering all 5 cases, on average, imatinib C_trough_ increased significantly by 70% during COVID-19 (95% CI: +42% to +99%), whereas norimatinib increased by 44% (95% CI: −3% to +90%; not significant). Patients 2 and 3 had no *CYP3A4* or *CYP3A5* variant potentially influencing imatinib metabolism. Patients 4 and 5 had genetic profiles consistent with an intermediate metabolizer status for CYP3A5 (CYP3A5*1/*3). Notably, case 4, with a more proficient *CYP3A5* genotype, presented the lowest increase in imatinib exposure. It is noteworthy that patient 4 also presented the *ABCB1* haplotype, deriving from the combination of *ABCB1* polymorphisms (rs1128503-1236C>T, rs1045642-3435C>T, and rs2032582-2677G>T/A), in the homozygous state. This haplotype was associated with a low functionality/protein expression of P-gp (11). The reduced activity/expression of P-gp at the intestinal level is potentially linked to a reduced drug plasma exposure, although to date no data confirm this association. Patient 5, also carrier of CYP3A5*1/*3, presented, on the other side, the *ABCG2* rs2231142-421C>A polymorphism in the homozygous variant status (AA), which was previously associated with higher imatinib plasma concentrations (9). AGP and CRP levels did not show a consistent trend with drug concentration, except in case 2 (CRP) and case 4 (AGP) (**Table 2**).

## 4 Discussion

In this work, we described for the first time the effect of mild COVID-19 on intra-patient variation in imatinib exposure (and related toxicity) in 5 patients with GIST. All patients exhibited increased plasma imatinib levels, peaking immediately after SARS-CoV-2 infection and remaining elevated for up to 6 months post-recovery. Some patients experienced temporarily reduced treatment tolerance.

Severe COVID-19 is known to affect drug metabolism, as evidenced by a prospective observational study which demonstrated an isoform-specific effect on the activity of 6 CYP450 enzymes. The infection was shown to decrease CYP3A, CYP1A2 and CYP2C19 while increasing CYP2B6 and CYP2C9 activity, with no significant effect on CYP2D6 (12). Numerous other studies indicate (or predict) that severe COVID-19 results in increased plasma levels and reduced hepatic clearance of drugs used to treat COVID-19 or other patients’ conditions (3, 13). Although the effect of severe COVID-19 in drug pharmacokinetics is well documented, information on mild COVID-19 is limited. The literature indicates that both mild and severe forms of the disease lead to alterations in circulating leukocyte subsets and cytokine secretion (14), which may similarly affect CYP enzymes activity. Accordingly, our findings highlight a statistically significant increase in patients’ imatinib exposure. Notably, we also observed an increase of norimatinib plasma levels with a metabolic ratio which remains almost stable during COVID-19 infection in 4/5 cases. We hypothesize that CYP enzyme suppression reduces the conversion of imatinib to norimatinib, leading to elevated imatinib levels. Contrary to expectations, norimatinib levels increased, likely due to disproportionate inhibition of its further metabolism, causing accumulation. A similar effect was observed in a study where ritonavir, a potent CYP3A4 inhibitor, did not significantly affect imatinib levels but increased norimatinib exposure by about 40%, approximately, the same increased observed in this study (15). The authors suggested that, unlike imatinib, which is metabolized through multiple pathways, norimatinib elimination is highly dependent on CYP3A4, making it more sensitive to acute inhibition. However, further dedicated studies will be requested to better clarify the overall basis of the observed increase in drug and metabolite exposure. Although specific data on COVID-19 are lacking, inflammation can affect cellular transporters such as P-glycoprotein, responsible for imatinib trans-cellular transport (16), that might contribute to increased imatinib exposure. Immunological dysfunction related to COVID-19 can persist for up to 8 months after mild to moderate SARS-CoV-2 infection (17). This suggests a potential similarly prolonged impact on drug metabolism, that is in line with our observation of higher imatinib and norimatinib concentrations persisting for up to 6 months in our patients.

To the best of our knowledge, this is the first study that analyzes the disease–drug interaction between mild COVID-19 and imatinib and norimatinib among cancer patients with GIST. A previous research focused on repurposing oral imatinib for severe COVID-19 because of its protective effects on the endothelial vascular barrier, which is compromised in COVID-19 patients (18). To assess potential exposure-response relationship within this new indication, total imatinib exposure was assessed in severe COVID-19 patients treated with the drug. Our results agree with those of this and other studies demonstrating that plasma imatinib C_trough_ in patients receiving the drug to treat COVID-19 was 2.3-fold higher than in patients without COVID-19 and receiving imatinib for cancer treatment (10).

COVID-19 is known to up-regulate acute-phase proteins such as CRP and AGP which can affect imatinib disposition (10). While AGP binds imatinib and can contribute in small part to its pharmacokinetic variability (19), our data do not show a clear correlation between AGP level and imatinib C_trough_ except in cases 1 and 4. Consistently with other studies, intra-patients variations in AGP did not correlate with the changes in imatinib C_trough_ (19). Regarding CRP, only case 2 showed levels above the normal range in all the samples tested, suggesting a baseline inflammatory burden heavier than those of the other patients, with a peak of CRP concentration in the first sample after COVID-19. This suggests an exacerbation of inflammation following COVID-19 infection, which later returned to the patient’s typical values in subsequent samples. However, this analysis had some limitations. Firstly, the markers were measured retrospectively on archival samples rather than freshly collected ones, possibly affecting the quality of the results. Secondly, most of the samples tested had CRP values below the assay’s lower limit of quantification (reported as <0.290 mg/mL). Consequently, we were unable to detect any variations even if they occurred. Lastly, the mild form of COVID-19 experienced by these patients may explain the unclear association between CRP and imatinib C_trough_ variation.

Notably, the patients with the highest increases in imatinib C_trough_ (cases 1, 3, and 5) also experienced increased imatinib-related toxicity. However, only case 5 reached a C_trough_ > 3000 ng/mL, associated with increased risk of toxicity. This may suggest that intra-patient variation in imatinib exposure, rather than its absolute level, might be important for the onset of toxicity. Moreover, elevated norimatinib levels, may have contributed to the increased toxicity in certain patients, also according to recent findings (20). This may support the possibility to consider the sum of imatinib and norimatinib C_trough_, similar to the approach used for sunitinib, as a more reliable marker of exposure than imatinib concentration alone in the clinical practice (21). Interestingly, CYP3A5*1/*3 genotype, corresponding to an increase in the patients metabolic efficiency, appeared to limit the rise in plasma drug exposure in one case (patient 4) who did not experienced toxicity. On the other side, the two patients with CYP3A5*1/*3 genotype had two different genetic backgrounds of the transporters. While patient 4 was bearing the *ABCB1* haplotype linked to a reduced drug intestinal transport and displayed a baseline exposure of 730 ng/mL, patient 5 was carrier of the *ABCG2* homozygous variant genotype, linked to a higher imatinib plasma concentrations, and displayed a baseline imatinib exposure of 2064 ng/mL.

Factors such as life-style habits (diet, cigarette smoking and alcohol intake) and co-medications may affect drug exposure. None of the patients included in this case series were smokers, but two were on multiple co-medications. Polypharmacy is common in adult patients with cancer and increases the risk of potential DDIs (22). Some of the drugs taken by the patients were substrates of CYP3A4, CYP2C19, CYP2D6, CYP2C8, and of the transporter BCRP. The effect of the COVID-19- cytokine storm on co-medications metabolism and transport could have potentially caused variations in patients’ exposure to these drugs with consequences on the toxicity profile, especially for drugs with overlapping toxicity spectra with imatinib. Although this was not observed in our study, the use of co-medications in GIST patients treated with imatinib should be carefully managed to avoid DDIs, as these can increase the risk of toxicity, particularly during COVID-19 infection.

## 5 Conclusion

We investigated the effect of mild COVID-19 on intra-patient fluctuation of imatinib and norimatinib exposure in GIST and observed an increase in their plasma levels that lasts up to 6 months, with a related decrease in treatment tolerability in 3/5 cases. This suggests that healthcare providers should be aware of these potential changes and consider monitoring drug levels more frequently in patients with mild COVID-19. This is especially important for patients on drugs with narrow therapeutic indices such imatinib and for those who have shown suboptimal tolerance to the treatment. These findings emphasize the importance of using an intensified pharmacological monitoring to detect variations in drug exposure that could increase the risk of toxicity.

## Data Availability

The original contributions presented in the study are included in the article/supplementary material, further inquiries can be directed to the corresponding author/s.
